# Psychosocial family factors and glycemic control among children aged 1-15 years with type 1 diabetes: a population-based survey

**DOI:** 10.1186/1471-2431-11-118

**Published:** 2011-12-20

**Authors:** Anne Haugstvedt, Tore Wentzel-Larsen, Berit Rokne, Marit Graue

**Affiliations:** 1 Faculty of Health and Social Sciences, Bergen University College, Norway; 2 Department of Public Health and Primary Health Care, University of Bergen, Norway; 3 Department of Pediatrics, Haukeland University Hospital, Bergen, Norway; 4 Centre for Clinical Research, Haukeland University Hospital, Bergen, Norway; 5 Centre for Child and Adolescent Mental Health, Eastern and Southern Norway, Oslo, Norway; 6 Norwegian Centre for Violence and Traumatic Stress Studies, Oslo, Norway

## Abstract

**Background:**

Being the parents of children with diabetes is demanding. Jay Belsky's determinants of parenting model emphasizes both the personal psychological resources, the characteristics of the child and contextual sources such as parents' work, marital relations and social network support as important determinants for parenting. To better understand the factors influencing parental functioning among parents of children with type 1 diabetes, we aimed to investigate associations between the children's glycated hemoglobin (HbA_1c_) and 1) variables related to the parents' psychological and contextual resources, and 2) frequency of blood glucose measurement as a marker for diabetes-related parenting behavior.

**Methods:**

Mothers (*n *= 103) and fathers (*n *= 97) of 115 children younger than 16 years old participated in a population-based survey. The questionnaire comprised the Life Orientation Test, the Oslo 3-item Social Support Scale, a single question regarding perceived social limitation because of the child's diabetes, the Relationship Satisfaction Scale and demographic and clinical variables. We investigated associations by using regression analysis. Related to the second aim hypoglycemic events, child age, diabetes duration, insulin regimen and comorbid diseases were included as covariates.

**Results:**

The mean HbA_1c _was 8.1%, and 29% had HbA_1c _≤ 7.5%. In multiple regression analysis, lower HbA_1c _was associated with higher education and stronger perceptions of social limitation among the mothers. A higher frequency of blood glucose measurement was significantly associated with lower HbA_1c _in bivariate analysis. Higher child age was significantly associated with higher HbA_1c _both in bivariate and multivariate analysis. A scatterplot indicated this association to be linear.

**Conclusions:**

Most families do not reach recommended treatment goals for their child with type 1 diabetes. Concerning contextual sources of stress and support, the families who successfully reached the treatment goals had mothers with higher education and experienced a higher degree of social limitations because of the child's diabetes. The continuous increasing HbA_1c _by age, also during the years before puberty, may indicate a need for further exploring the associations between child characteristics, context-related variables and parenting behavior such as factors facilitating the transfer of parents' responsibility and motivation for continued frequent treatment tasks to their growing children.

## Background

The Diabetes Control and Complication Trial confirmed the significant association between poor glycemic control and higher risk of long-term complications among adolescents with type 1 diabetes [1]. Since then, insulin treatment and technologies for insulin delivery have improved and international guidelines for managing diabetes among children and adolescents have been established. Although some studies have reported improved glycated hemoglobin (HbA_1c_) among children in recent decades [2-4], no unambiguous evidence indicates that technical and medical progress has substantially improved glycemic outcomes [5,6]. Many children and adolescents still do not achieve HbA_1c _less than 7.5% as recommended by the International Society for Pediatric and Adolescent Diabetes (ISPAD) guidelines [7].

Several studies have highlighted the importance of not only considering the effects of medical and technical factors but also psychosocial family factors for glycemic outcomes among children with type 1 diabetes. The results of studies focusing on associations between psychosocial factors on glycemic control are, however, mixed. In addition, most results are based on small sample sizes and considerable variation in the instruments used to assess psychosocial variables.

Sherifali & Ciliska [8] claimed that most of the parenting research literature as related to children with diabetes lacks a conceptualization of the determinants influencing parental functioning. They suggested Jay Belsky's determinants of parenting model as a conceptual framework to guide future research on parenting children with diabetes. Belsky [9] stated that most parenting research has focused on the characteristics and consequences of parenting. By developing the determinants of parenting model Belsky drew attention to the determinants of individual differences in parenting. The model emphasizes 1) the parents' personal psychological resources, 2) the characteristics of the child and 3) contextual sources of stress and support as three important domains influencing the parenting process and subsequently the child's development. The contextual sources of stress and support include work, marital relations and social network support [9].

Caring for a child with diabetes requires continual sensitive adaptation to the child's growing and stage of development. Belsky [9] discussed what kind of personal psychological resources are needed to provide developmentally flexible and growth-promoting care. As part of the answer, Belsky claimed that previous research has provided some support for links between parents' mental well-being and their parental functioning. In accordance, the Hvidøre Study Group on Childhood Diabetes [10] has demonstrated a positive association between parents' experience of well-being and glycemic control among children with diabetes. Subjective well-being has been reported to be facilitated by a person's trait of optimism, which has been shown to strongly protect adults who have experienced stressful life events such as the illness of a family member [11]. Based on this, it would be of interest to further examine the relationship between glycemic control among children with type 1 diabetes and variables related to the parents' life orientation regarding optimism as a marker for competent parental functioning for parents' caring for a child with type 1 diabetes.

Belsky's model emphasizes how contextual sources of stress and support such as social support, work and marital relations influence both parents' psychological resources and how they parent [9]. Sullivan-Bolyai et al. [12] have described how social support enhances mothers' abilities to cope with the demanding daily treatment tasks related to diabetes treatment in a child. Thus, the associations between social support and glycemic control should be further explored. The study of Sullivan-Bolyai further reported that mothers of children with type 1 diabetes had lower employment status than mothers in a control group, with the additional responsibility because of the child's diabetes as an explanatory factor. Although the association between employment status and glycemic control has not been fully explored, fathers' higher education level has been reported to be associated with better glycemic control among children with diabetes [13].

Parents living together have previously been stated as a robust determinant for lower HbA_1c _among adolescents with type 1 diabetes [10]. According to Belsky's model the parents' satisfaction with the marital relationship may also be important for how families handle the daily challenges related to a child's diabetes treatment. A study among 109 children 8-18 years old and one of the parents showed that family functioning, the families' adherence to diabetes treatment, family structure, the child's age and age at diagnosis explained 49% of the variation in HbA_1c _[14].

An important part of parenting children with type 1 is the frequent daily treatment tasks required. Helgeson [15], Ziegler [16] and others have shown that the daily frequency of blood glucose measurement is correlated with better glycemic outcomes among children and adolescents with type 1 diabetes. Frequent measurement helps the parents and the child to adjust the insulin treatment and/or adjust dietary behavior. In addition, more frequent blood glucose measurement has been claimed to be a potential marker for good adherence to the diabetes management behavior [17]. Transferred to Belsky's conceptual framework, the frequency of blood glucose measurement may be a marker for good quality of care related to diabetes-specific parenting behavior and appropriate daily management of the child's diabetes.

Based on previous research and inspired by the Belsky's determinants of parenting model the objectives of our study were 1) to examine associations between glycemic control among children with type 1 diabetes and variables related to the parents' personal psychological resources (optimistic life orientation) and contextual sources of stress and support (social support, work and education and marital relations) and 2) to examine the association between glycemic control among the children and the frequency of blood glucose measurement as a marker for good quality of care related to the diabetes-specific parenting behavior. We hypothesized:

that an optimistic life orientation, higher parental education, higher degree of employment, two-parent status or higher perceived satisfaction with the marital relationship, higher degree of social support or less perceived social limitation because of the child's diabetes would be associated with lower HbA_1c _among children with type 1 diabetes; and.

that high frequency of blood glucose measurement would be associated with lower HbA_1c _when controlled for important child characteristics: frequency of problematic hypoglycemic events, the child's age, duration of diabetes, insulin regimen and comorbid diseases.

## Methods

In this population-based study we invited the parents of 161 children in Hordaland County, Norway to participate. All the children had type 1 diabetes for more than 3 months and were ≤ 15 years old. We sent a study information sheet and identical questionnaires for mothers and fathers by mail to the parents. We informed the parents that completed and returned questionnaires would be considered informed consent and that data on current HbA_1c _and insulin regimen would be collected from medical records. The Western Norway Regional Medical and Health Research Ethics Committee and the Norwegian Social Science Data Services approved this procedure and approved an anonymous nonrespondent analysis including HbA_1c _values in addition to age, sex and diabetes duration. The study was performed according to the Declaration of Helsinki.

### Instruments

We used standardized questions and standardized instruments recommended by the Norwegian Institute of Public Health to collect on demographic variables from the parents. We collected data on routines for blood glucose measurement, hypoglycemic events and comorbid diseases among the children from both mothers and fathers. The reports from a child's mother and father agreed close to 100%. In the analysis, we primarily used data from the mother if they were available; if not, we used data from fathers. We used the DCA-2000 (Bayer, Elkhart, IN, USA) for the HbA_1c _analysis (normal range 4.5-6.1%).

The questionnaire included several recognized generic scales (Additional file 1). We used the Life Orientation Test to collect data on the parents' trait of optimism or pessimism as part of the parents' psychological resources. The Life Orientation Test is a self-report instrument with 8 items (such as "In uncertain times I usually expect the best") [18,19]. A sum score (range 0-32) is obtained by summing the item scores. Higher scores indicate a more optimistic life orientation. Cronbach's alpha for the Life Orientation Test in this study was 0.81 for the mothers and 0.74 for the fathers. This is comparable with previous reports [18,19].

Concerning contextual sources of stress and support, we used the Oslo 3-item Social Support Scale sum score to measure the parents' experience of social network support. The items in the scale include 1) number of confidants, 2) sense of concern or interest from other people and 3) sense of support from neighbors [20,21]. WHO recommends the Oslo 3-item Social Support Scale for use in health surveys, and a sum index ranging from 3 to 14 is derived by adding the scores. A higher score indicates more social support [20]. Cronbach's alpha for the Oslo 3-item Social Support Scale in this study was 0.71 for the fathers and 0.55 for the mothers. The low Cronbach's alpha for the mothers in this study caused an exclusion of the scale in the analysis of mother-reported data. In addition to the Oslo 3-item Social Support Scale, we added a single question exploring the parents' experience of social limitation because of the child's diabetes, with three categories (none or slight, somewhat or strong experience).

To assess satisfaction with the marital relationship, we used the Relationship Satisfaction Scale, with five statements on satisfaction with the marital relationship (such as "I am very happy in my marital relationship"). The items are rated on a six-point Likert scale, and the scale score is calculated by adding the item scores [22]. Cronbach's alpha (0.88 for fathers and 0.89 for mothers) showed good internal consistency for the Relationship Satisfaction Scale in this study comparable with previous reports [22].

Overall, few data related to the parents' answers on the Life Orientation Test, the Oslo 3-item Social Support Scale and the Relationship Satisfaction Scale were missing. We performed missing substitution to calculate the scale scores by inserting the mean when at least 4 of 8 (Life Orientation Test), 2 of 3 (Oslo 3-item Social Support Scale) or 3 of 5 (Relationship Satisfaction Scale) items were answered. We excluded one father from the Life Orientation Test and one mother and two fathers from the Relationship Satisfaction Scale because of too many missing values.

### Statistical analysis

We carried out statistical analysis using SPSS version 17.0 (SPSS Inc., Chicago, IL, USA). We performed linear regression to test the hypotheses of associations between the explanatory variables and the child's HbA_1c _as dependent variable. The sample size and the fact that some of the variables mutually excluded each other (marital status and satisfaction with the marital relationship) limited the possibility of including all explanatory variables in one multiple regression analysis. For comparison, we started with performing separate regression analyses for each explanatory and control variable included in the hypotheses. Further, we performed three multiple regression analyses. For the first hypothesis (including life orientation, social support, perceived social limitation, employment status, education and marital status), we performed one analysis for mothers and one for fathers. The Relationship Satisfaction Scale was not included in these multiple regression analyses, as this would have excluded single parents. For the second hypothesis (including the frequency of blood glucose measurement and the control variables frequency of problematic hypoglycemic events, the child's age, duration of diabetes, insulin regimen and comorbid diseases), we performed one multiple regression analysis. Additionally, we explored the relationship between age and HbA_1c _by performing a scatterplot and HbA_1c _by age groups by performing ANOVA.

## Results

### Clinical and demographic variables

The parents of 115 children returned the study questionnaire (response rate 72%). The participants were 103 mothers and 97 fathers. Both parents answered the questionnaire in 85 cases, only the mother in 18 cases and only the father in 12 cases.

The 115 children had a mean duration of diabetes of 3.9 years (SD 2.9, range 0.3-14.2). Five children had diabetes for less than 1/2 a year, with a minimum of 3.5 months, and additional 12 had diabetes for less than 1 year. Table 1 shows the children's age and HbA_1c_, use of an insulin pump, frequency of daily blood glucose measurement and frequency of problematic hypoglycemic events in the past year overall and by age group. HbA_1c _did not differ significantly between girls (HbA_1c _= 8.03%) and boys (HbA_1c _= 8.17%) (*P *= 0.468). All children received intensive insulin treatment with either an insulin pump or three or more insulin injections per day. Blood glucose was measured frequently (Table 1), and the parents of 27% of the children reported measurement at night at least once weekly. However, only 29% (*n *= 33) of the children had HbA_1c _≤ 7.5% as recommended by ISPAD [7].

**Table 1:** Characteristics of 115 children (aged 1-15 years) with type 1 diabetes by age group

	*n *(*%*)	Mean (range)	SD
Mean age (years)		10.6 (1.6-15.9)	3.6
**Age groups**			
1-5 years	13 (11)		
6-11 years	57 (50)		
12-15 years	45 (39)		
**HbA_1c _(%)**		8.1 (5.3-11.7)	1.0
1-5 years		7.2 (5.3-8.2)	0.9
6-11 years		8.0 (6.1-10.3)	0.8
12-15 years		8.4 (6.4-11.7)	1.1
**Insulin pump**	50 (43)		
1-5 years	5 (39)		
6-11 years	23 (40)		
12-15 years	22 (49)		
Blood glucose measurements per day*, 4-6/≥ 7	64 (56)/36 (31)		
1-5 years	7 (54)/6 (46)		
6-11 years	31 (54)/21 (37)		
12-15 years	26 (60)/9 (21)		
Problematic hypoglycemic events in the past year*, ≥ 7	26 (23)		
1-5 years	4 (33)		
6-11 years	11 (20)		
12-15 years	11 (25)		

*The mothers' reports were used if available; if not, data from fathers were used (the data showed close to 100% agreement between mothers' and fathers' reports on these items).

Nearly all parents (97%) were of Norwegian ethnicity. The mothers' mean age was 39.6 ± 5.7 years and the fathers' 42.6 ± 6.4 years. Fifteen percent of the mothers and 12% of the fathers reported single-parent status. About half the parents (45% of the mothers and 55% of the fathers) reported education at university or university college level. Five percent had not graduated from upper-secondary school. Of the fathers, 92% reported full-time employment, whereas only 37% of the mothers reported this. Many mothers (45%) reported part-time employment. Of the fathers, 45% reported perceived social limitation somewhat or strongly because of the child's diabetes. Of the mothers, 48% reported the same.

The parents of 46 (28%) children did not return the study questionnaire. The HbA_1c _did not differ significantly between the children of nonrespondents and respondents, but the children of nonrespondents were 1.7 years older (*P *= 0.005) and the duration of diabetes was 1.3 years longer (*P *= 0.016) than that of the children of respondents.

### Characteristics of parents and child *HbA*_*1c*_

Regarding the first hypothesis of associations between the children's HbA_1c _and the parents personal psychological resources and contextual sources of stress and support, the analyses indicated no significant associations between the children's HbA_1c _and the parents' trait of optimism or pessimism as measured by the Life Orientation Test, the fathers' experience of social support (Oslo 3-item Social Support Scale) and the parents' satisfaction with the marital relationship (Relationship Satisfaction Scale) (Table 2). Further, the children's glycemic control was not significantly associated with the parents' employment status or marital status. However, a higher education level among the mothers was significantly associated with better glycemic control in their children in both bivariate and multivariate analysis (Table 2). In addition, and in contrast to what we hypothesized, lower HbA_1c _among children was significantly associated with strong versus no or slightly perceived social limitation among the mothers in the multiple regression analysis. The same trend was identified between HbA_1c _and the fathers' experience of social limitation, but this association was not statistically significant (Table 2). Strong experience of social limitation because of the child's diabetes was reported by 20% of the mothers and 17% of the fathers in the study.

**Table 2:** Bivariate and multiple linear regression analysis for mother- and father-related variables associated with HbA_1c _among children (*n *= 115) with type 1 diabetes

	HbA_1c_			
	**Bivariate regression**		Multiple regression*	
	**Coefficient**	***P***	**Coefficient**	***P***

**Mother-related variables**				
Life Orientation Test	0.01	0.756	-0.02	0.388
Education - university or university college vs. not	-0.47	0.016^†^	-0.58	0.008†
Employment status				
Working part time vs. full time	-0.23	0.329	-0.36	0.116
Unemployed vs. working full time	-0.16	0.588	-0.52	0.079
Oslo 3-item Social Support Scale ^§^	-	-	-	-
Experience of social limitation				
Somewhat vs. none or slight	-0.08	0.733	-0.04	0.853
Strong vs. none or slight	-0.43	0.116	-0.62	0.022^†^
Marital status - single mother vs. not	0.25	0.414	0.32	0.268
Relation Satisfaction Scale	-0.01	0.540	-	-
**Father-related variables**				
Life Orientation Test	0.00	0.984	0.00	0.849
Education - university or university college vs. not	-0.16	0.484	-0.07	0.763
Employment status				
Working part time vs. full time	-0.12	0.912	-0.38	0.723
Unemployed vs. working full time	0.30	0.463	0.41	0.337
Oslo 3-item Social Support Scale	0.00	0.995	0.01	0.884
Experience of social limitation				
Somewhat vs. none or slight	-0.47	0.054	-0.50	0.063
Strong vs. none or slight	-0.37	0.213	-0.33	0.314
Marital status - single father vs. not	0.48	0.169	0.64	0.111
Relation Satisfaction Scale	-0.01	0.626	-	-

*One multivariate analysis for mother-related variables (*R*^2 ^= 0.17) and one for father-related variables (*R*^2 ^= 0.07).

^†^*P *< 0.05.

^§ ^The Oslo 3-item Social Support Scale was excluded in analyses among the mothers because of low Cronbach's alpha (0.55).

### Blood glucose measurements, child characteristics and HbA_1c_

In relation to the second hypothesis of associations between frequency of blood glucose measurement as a marker for good quality of care related to the diabetes-specific parenting behavior and HbA_1c_, we identified a significant association between ≥ 7 blood glucose measurements per day and lower HbA_1c _in the bivariate analysis (Table 3). This significant association did not appear in the multiple regression analysis. Only the child's age was significant positively associated with HbA_1c _in the multiple regression analysis related to the second hypothesis (Table 3).

**Table 3:** Bivariate and multiple linear regression analysis of frequency of blood glucose measurement and child-related control variables associated with HbA_1c _among children (*n *= 115) with type 1 diabetes

	HbA_1c_			
	**Bivariate regression**		Multiple regression*	

	**Regression** **coefficient**	***P ***	**Regression** **coefficient**	***P***

Blood glucose measurement per day				
4-6 times versus ≤ 3 times	-0.44	0.155	-0.17	0.586
≥ 7 times versus ≤ 3 times	-0.79	0.019^†^	-0.28	0.403
Problematic hypoglycemia in the past year				
1-2 episodes versus 0 episodes	0.07	0.793	-0.07	0.774
3-6 episodes versus 0 episodes	0.04	0.896	0.22	0.403
≥ 7 episodes versus 0 episodes	-0.12	0.662	-0.02	0.936
Age	0.13	< 0.001^†^	0.12	< 0.001^†^
Duration of diabetes	0.11	0.001^†^	0.01	0.775
Insulin pump - yes versus no	0.41	0.033^†^	0.32	0.109
Comorbid disease - yes versus no	0.23	0.292	0.21	0.329

*R^2 ^(for the multivariate analysis) = 0.25.

^†^*P *< 0.05.

**Figure 1:**
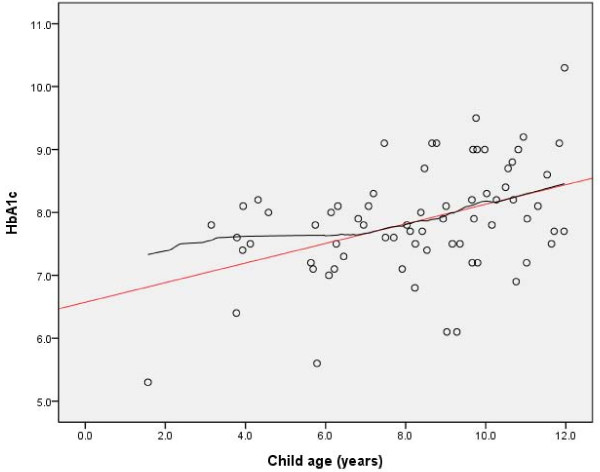
**HbA_1c _by child age among children ≤ 11 years (*n *= 70)**. A scatter plot of the relationship between age and HbA_1c_.

After performing the analyses related to the two hypotheses we explored further the association between the children's age and HbA1c. The exploratory analysis of variance showed significant differences in mean HbA_1c _between the age groups 1-5 years, 6-11 years and 12-15 years (*P *= 0.004). Higher age group indicated higher HbA_1c _levels (Table 1). The scatterplot of HbA_1c _by child age, including only the children < 12 years of age, showed a close to linear relationship between higher age and higher HbA_1c _between 7 and 12 years of age. Figure 1 illustrates the relationship by both a loess and a linear line.

## Discussion

The results of this population-based study highlight the importance of revealing the factors associated with glycemic control among children with type 1 diabetes since only 29% of the children in the study achieve the recommended treatment goals with HbA_1c _≤ 7.5% [7]. According to the Belsky's determinants of parenting model, we did not identify any significant associations between contextual sources of stress and support related to the fathers and the children's glycemic control, although we have previously reported a quite similar experience of diabetes-related burden among the mothers and the fathers in this population-based study [23]. In accordance with previous reports [24], the results support a presumption of the mother as most often the primary caregiver of children with type 1 diabetes.

### Perceived social limitation and maternal education

The social limitation experienced by the parents in our study appeared to be associated with lack of options to transfer the responsibility for the child to someone else. Sullivan-Bolyai et al. [12] found that only 36% of the mothers of children with diabetes reported having access to child care versus 83% of the mothers in a control group. There seems to be a substantial need for help in building and educating a network around the families: a network of people the parents easily can trust and to whom the responsibility for the child can be delegated occasionally. The association between perceived social limitation and better glycemic control may be interpreted as ambitious mothers who have high aims for the child's diabetes treatment. Families who are coping well may be able to successfully integrate the disease into their daily routines, but these efforts have certain costs, since they might spend much of their total available energy on treatment issues related to the child with diabetes. High ambitions may also create difficulty in transferring responsibility to other people. It is demanding to be among the best, and health care providers should not ignore severe experiences of burden and distress that may require somewhat different support and guidance compared with the needs of parents of children with poor glycemic control. Further research is needed to enhance overall knowledge on the effects of social support and assistance for the parents of children with type 1 diabetes.

The effects of mothers' education may indicate that current diabetes management plans require knowledge and resources that more highly educated people have to a higher degree than other people. Diabetes teams therefore face challenges in fitting treatment plans for all families. Diabetes affects all strata of society, and support and interventions need to be adjusted for contextual factors related to families with various psychosocial and sociodemographic backgrounds. Nevertheless, one could question whether today's diabetes treatment plans are too complicated and too time-consuming for some families. Most families do not achieve treatment goals despite many daily treatment tasks.

### Blood glucose measurements and the child's age

An important part of parenting children with type 1 diabetes is the high number of blood glucose measurements required to achieve treatment goals. In this study, the parents reported a high frequency of measurement, and it appeared that more frequent measurement was associated with a significant decrease in HbA_1c _(Table 3). Higher frequency of blood glucose measurement has also previously been shown to be associated with lower HbA_1c _[3,15,16]. In accordance with Helgeson et al. [15] and Ziegler et al. [16], we identified the highest frequency of blood glucose measurement in the youngest age group (1-5 years) (Table 1). The explanation could be that the parents of the youngest children are more motivated for frequent treatment tasks than the parents of older children or older children themselves.

The association between higher child age and higher HbA_1c _may also be explained by factors related to transferring responsibility, knowledge and motivation from the parents to the child or significant others in the child's social network. It is well known that HbA_1c _is higher among adolescents than among younger children [4,16]. During puberty, increasing HbA_1c _can partly be explained by the additional challenges related to hormonal changes and reduced insulin sensitivity during this period. Previous research studies have not highlighted and discussed the explanations for increasing HbA_1c _among children 7-12 years old, but the reasons might be other than physical ones. As a child grows, the roles change, and the responsibility for daily treatment tasks is transferred from the parents to the child. Too early transfer of responsibility has previously been reported to be associated with poor glycemic control [25]. Our findings may support this. Children can manage technical treatment tasks quite early, but the age at which they can take responsibility for the medical decisions is probably much higher and varies from child to child. In accordance, parents need to receive better-adjusted guidance in the process of transferring responsibility and motivation for treatment tasks to their child. Interventions for both children and significant others are needed to increase knowledge, ability and motivation for the important treatment tasks required, also when the parents are not present.

### Strengths and limitations

The study has strengths and limitations. The cross-sectional design presents limitations, especially concerning the impossibility of identifying causality. However, the inclusion of parents of all children with type 1 diabetes up to 16 years of age in a population-based study is strength of the study. Further, to our knowledge no previous studies have included both the mother and the father of such a large number of children with type 1 diabetes in the same study.

While HbA_1c _may be less stable shortly after diabetes onset one could suggest excluding the children with less than 1 year duration of diabetes from the analyses. A sensitivity analysis performed in this study did, however, not indicate substantially different results when excluding the ones with less than one year duration of diabetes. Mean HbA_1c _among the 98 children with ≥ 1 year duration of diabetes was 8.2% compared with 8.1% among the total group of 115 children.

Life Orientation Test, Oslo 3-item Social Support Scale and Relationship Satisfaction Scale are valid and recommended instruments. However, they might be too general to reveal the specific contextual factors or psychological resources of importance to achieve satisfactory diabetes treatment outcomes. The somewhat weak Cronbach's alpha for the mothers' Oslo 3-item Social Support Scale scores may be a result of few items in the Scale or a result of the mothers' mixture of perceived general social support and disease-specific social support.

## Conclusions

Only 29% of the families in the study achieved HbA_1c _≤ 7.5% in their child with type 1 diabetes. According to Belsky's determinants of parenting model, the study identified some important associations between contextual sources, the characteristics of the child and parenting. The families who successfully reached diabetes treatment goals had more highly educated mothers and experienced more social limitations because of the child's diabetes than the parents of children with poor glycemic control. Based on these results, one may question whether modern diabetes treatment plans are too complicated or not well adapted for each individual family. The increasing HbA_1c _by age, also during the childhood years well before puberty, may indicate a need for further exploring the associations between child characteristics and parenting behavior such as factors facilitating the transfer of parents' responsibility and motivation for continued frequent treatment tasks to their growing children.

## Competing interests

The authors declare that they have no competing interests.

## Authors' contributions

AH researched data, wrote the manuscript, contributed to discussion and reviewed and edited the manuscript. TW-L researched data, contributed to discussion and reviewed and edited the manuscript. BR researched data, contributed to discussion and reviewed and edited the manuscript. MG researched data, contributed to discussion and reviewed and edited the manuscript. All authors read and approved the final manuscript.

## Pre-publication history

The pre-publication history for this paper can be accessed here:

http://www.biomedcentral.com/1471-2431/11/118/prepub

## Supplementary Material

Additional file 1 **Questionnaire**. The scales included in the study. Click here for file
