# Supplementary material for: Psychosocial family factors and glycemic control among children aged 1-15 years with type 1 diabetes: a population-based survey
Source: BMC Pediatr. 2011 Dec 20;11:118. doi: 10.1186/1471-2431-11-118 (PMC3282662; doi:10.1186/1471-2431-11-118)
Supplement: Additional file 1 — Questionnaire. The scales included in the study. [file 1471-2431-11-118-S1.DOC]

**Questionnaire**

The scales included in the study among the mothers and fathers

of 115 children with type 1 diabetes (<16 years)

**Life orientation test** (Ref. 1)

|  |  | **1**  Strongly disagree | **2**  Disagree | **3**  Agree | **4**  Strongly agree |
| --- | --- | --- | --- | --- | --- |
| 1. | In uncertain times, I usually expect the best. |  |  |  |  |
| 2. | If something can go wrong with me, it will. |  |  |  |  |
| 3. | It's easy for me to relax. |  |  |  |  |
| 4. | I am always optimistic about my future. |  |  |  |  |
| 5. | I hardly ever expect things to go my way. |  |  |  |  |
| 6. | Things never work out the way I want them to. |  |  |  |  |
| 7. | I'm a believer in the idea that "every cloud has a silver lining." |  |  |  |  |
| 8. | I rarely count on good things happening to me. |  |  |  |  |

**Relationship Satisfaction Scale** (Ref. 2)

(*Complete if you are in a relationship).*

|  |  | **1**  Strongly  agree | **2**  Agree | **3**  Some-what  agree | **4**  Some-  what  disagree | **5**  Disagree | **6**  Strongly disagree |
| --- | --- | --- | --- | --- | --- | --- | --- |
| 1. | My partner and I have problems in our relationship. |  |  |  |  |  |  |
| 2. | I am very happy in my relationship. |  |  |  |  |  |  |
| 3. | My partner is generally understanding. |  |  |  |  |  |  |
| 4. | I am very happy in my relationship. |  |  |  |  |  |  |
| 5. | We agree on how children should be brought up. |  |  |  |  |  |  |

**Oslo 3 item Social Support Scale** (Ref. 3-4)

1. How many people are so close to you that you can count on them if you have serious problems?

(Include also relatives)

None

1-2 persons

3-5 persons

More than 5 persons

2. How much concern do people show in what you are doing?

A lot

Some

Little

None

Uncertain

3. How easy can you get help from neighbours if you should need it?

Very easy

Easy⁭

Possible

Difficult

Very difficult

**Single question regarding social limitation because of the child’s diabetes:**

- Do you experience social limitations because of the child’s diabetes?

Not at all

To slight degree

To somewhat degree

To strong degree

To very strong degree

**References**

1. Thuen F, Rise J. **Psychological adaptation after marital disruption: the effects of optimism and perceived control.** *Scand J Psychol* 2006, **47**:121–128.
2. Dyrdal GM, Røysamb E, Nes RB, Vittersø J. **Can a happy relationship predict a happy life? A population-based study of maternal well-being during the life transition of pregnancy, infancy and toddlerhood.** *J Happiness Stud* 2010, doi: 10.1007/s10902-010-9238-2.
3. Meltzer H. **Development of a common instrument for mental health.** In *EUROHIS: Developing Common Instruments for Health Surveys*. Edited by Nosikov A, Gudex C. Amsterdam, IOS Press (on behalf of WHO), 2003.
4. Dalgard OS, Dowrick C, Lehtinen V, Vazquez-Barquero JL, Casey P, Wilkinson G, Ayuso-Mateos JL, Page H, Dunn G; Odin Group. **Negative life events, social support and gender differences in depression.** *Soc Psychiatry Psychiatr Epidemiol* 2006, **41**:444–451.
